# Spontaneous full thickness macular hole development and closure in a patient with nucleus dislocation due to hypermature cataract: a case report

**DOI:** 10.3325/cmj.2020.61.366

**Published:** 2020-08

**Authors:** Sanja Petrovic Pajic, Xhevat Lumi, Petra Schollmayer, Marko Hawlina

**Affiliations:** 1 Eye Hospital, University Medical Center Ljubljana, Ljubljana, Slovenia; 2 Clinic for Eye Diseases, Clinical Center of Serbia, Belgrade, Serbia

## Abstract

Spontaneous posterior capsule rupture with lens-nucleus dislocation is a very rare entity, as is the development and spontaneous closure of a full thickness macular hole (FTMH) after vitrectomy. The occurrence of these two entities in one eye has not been previously described. A 79-year-old woman was referred because of the right eye intermittent pain and progressive visual loss. Best corrected visual acuity (BCVA) with correction for aphakia was 20/20. Intraocular pressure was normal with therapy. The cornea, anterior chamber, and vitreous were clear. Gonioscopy was normal. The capsular bag was clear, with rolled-up anterior and posterior lens capsule, and the nucleus dislocated in the vitreous. As surgery waiting time was prolonged due to administrative problems, the patient’s intraocular pressure (IOP) increased and cystoid macular edema (CME) with lamellar macular hole developed. The patient underwent pars plana vitrectomy with endophacofragmentation and epiretinal membrane peeling. Postoperative optical coherence tomography was normal, BCVA was 20/40, and IOP was normal with topical therapy. One month after surgery, the eye was without signs of inflammation and IOP started rising in spite of maximum therapy. CME reoccurred and progressed to a FTMH, which started closing spontaneously in one month. One year after surgery, IOP normalized and FTMH closed completely. A dislocated crystalline lens in a quiet eye with normal BCVA, which rapidly developed into intractable glaucoma and FTMH, is an unusual finding. The deterioration was followed by spontaneous IOP normalization and macular hole closure. Such unexpected disease course, suggesting a possible autoimmune reaction, has not yet been described.

Spontaneous posterior capsule rupture with lens-nucleus dislocation is a very rare entity. A few such cases have been described – in hypermature senile cataract (HMSC) (1), posterior polar cataracts (2), pseudoexfoliation (PEX) syndrome (3), Marshall syndrome, and after electrical injury (4). We report a case of a spontaneous nucleus dislocation with unexpected development during a follow up of two and a half years.

## Case report

**Figure 1:**
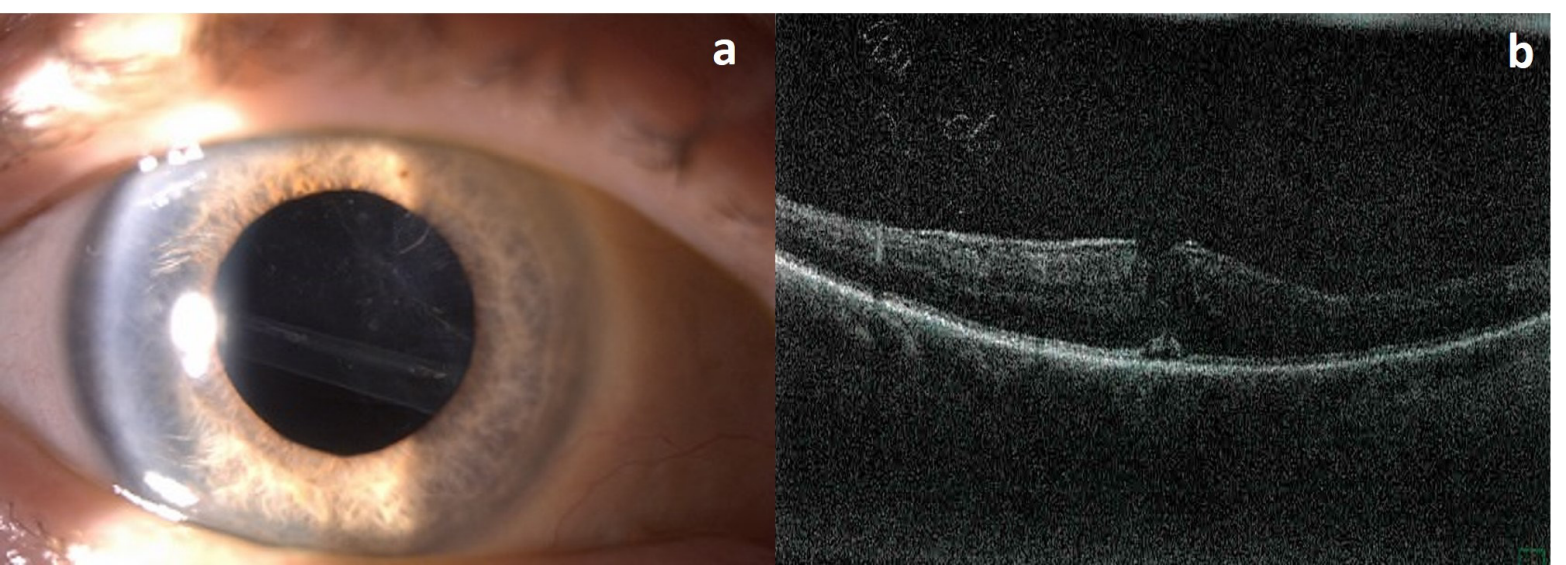
(**A**) Anterior segment on admission: anterior chamber without signs of inflammation, clear cornea and capsular bag without the remnants of the cortex, ruptured and rolled-up anterior and posterior lens capsule leaf, the nucleus dislocated into the vitreous cavity. (**B**) Optical coherent tomography on admission showed epimacular membrane.

**Figure 2:**
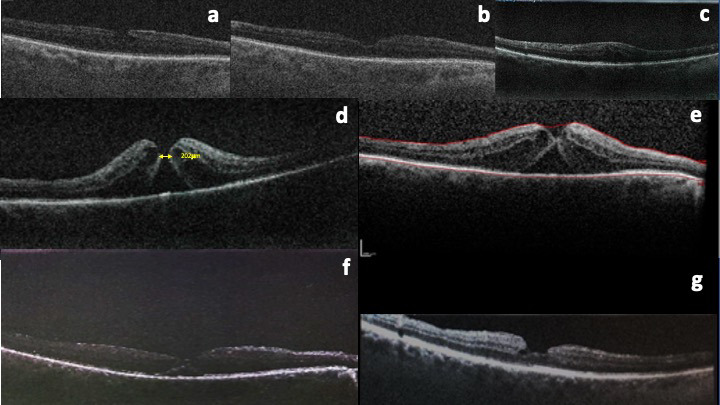
Optical coherent tomography images (**A**) Before surgery – lamellar hole, intraocular pressure (IOP) 20 mm Hg with topical therapy. (**B**) The second postoperative day – epimacular membrane completely removed and lamellar hole closed. (**C**) One month after surgery – cystoid macular edema and high IOP under topical therapy. (**D**) Three months after surgery – IOP 24 mm Hg despite maximum topical and oral therapy, full thickness macular hole. (**E**) Four/five months after surgery – the beginning of macular hole closure and IOP normalization. (**F**) Six months after surgery – almost complete spontaneous closure of the macular hole. (**G**) Year and a half after surgery – complete closure of the macular hole with a small intra-retinal cyst.

A 79-year-old woman was referred to Eye Hospital Ljubljana in February 2017 because of intermittent pain and progressive visual loss in the right eye due to dislocated nucleus and high intraocular pressure (IOP), which was soon reduced to normal values with topical anti-glaucomatous therapy. The patient reported no history of trauma or eye diseases. She had uneventful cataract surgery on the left eye 13 years previously, when she was informed about the existence of cataract on the right eye. General medical history was insignificant. On admission, right eye best corrected visual acuity (BCVA) with aphakic correction was 20/20, IOP was normal with topical therapy. The cornea, anterior chamber, and vitreous were clear. Gonioscopy was normal, with no signs of PEX. The capsular bag was clear without cortex, with a rolled-up anterior and posterior lens capsule (Figure 1A), and the nucleus dislocated in the vitreous at the equator at 6 o’clock. The optic disc was normal. Optical coherent tomography (OCT) showed an epimacular membrane (Figure 1B). Due to administrative problems, the surgery was postponed for three months. During this time, the patient’s IOP increased, BCVA decreased to 20/200 Snellen, and cystoid macular edema with lamellar macular hole developed (Figure 2A). The patient underwent pars plana vitrectomy (PPV) with endophacofragmentation of the extremely hard nucleus and epiretinal membrane peeling. At this stage, the eye was left aphakic. Postoperatively, macular morphology was normal (Figure 2B), BCVA was 20/40, and the IOP normalized with topical therapy. One month after surgery, IOP started rising again and CME reoccurred (Figure 2C) even though the eye was without signs of inflammation. Three months postoperatively, IOP was high despite maximum topical and oral therapy, and a 202-micron full thickness macular hole (FTMH) developed (Figure 2D). The patient was scheduled for surgery, but in two months FTMH started to close spontaneously, and IOP decreased to normal values (Figure 2E). At postoperative month 6, the hole was almost closed (Figure 2F), with complete closure a year and a half after surgery (Figure 2G). IOP remained normal with local anti-glaucoma therapy, and BCVA improved to 20/30 with aphakic correction (Table 1).

**Table 1:** Timeline of the disease course and interventions*

Date	Relevant medical history	
2005	Left eye cataract surgery and diagnosis of cataract on the right eye	
	**Summaries from the initial and follow-up visits**	**Interventions and therapy**
February 2017	VA decrease and IOP rise	Topical anti- glaucoma therapy
March 2017	IOP normalization; VA with aphakic correction 20/20. Ruptured and rolled-up lens capsule without cortex, hard lens nucleus dislocated into the vitreous cavity; no signs of inflammation. Epiretinal membrane in the macula.	Topical anti-glaucoma therapy. Suggested immediate vitrectomy for lens dislocation, but due to administrative problems (foreign health insurance), the patient had to wait for three months
June 2017	IOP rise with medication, CME and lamellar macular hole development	Topical and systemic anti-glaucoma therapy
July 2017	Patient admitted for surgery; high IOP with therapy, lamellar hole and epiretinal membrane	Vitrectomy with internal limiting membrane peeling
First postoperative day	Macular hole closure and IOP normalization	
Second postoperative day	Patient discharged, no signs of inflammation; BCVA 20/40	Topical anti-glaucoma therapy
August 2017 – one month after surgery	IOP rise and CME development, no signs of inflammation	
October 2017 – three months after surgery	IOP rise and full thickness macular hole (FTMH) development, VA drop, no signs of inflammation	Topical and systemic anti-glaucoma therapy; vitrectomy scheduled but due to administrative problems postponed for a month
November 2017	Patient admitted for surgery; lower IOP with therapy, beginning of the spontaneous closure of FTMH	Topical anti-glaucoma therapy, vitrectomy cancelled
November 2018	Spontaneous closure of FTMH and normalization of IOP, BCVA 20/30	Topical anti-glaucoma therapy
November 2019	Closed macular hole, BCVA 20/30, IOP 20 mm Hg	Topical anti-glaucoma therapy

*BCVA – best corrected visual acuity; VA – visual acuity; IOP – intraocular pressure; CME – cystoid macular edema; OCT – optical coherent tomography.

## Discussion

The mechanism of capsular rupture in our patient remains unknown. Posterior lenticonus cataract is an unlikely explanation as the anterior and posterior leaves of the capsule were rolled up, suggesting that the rupture was at the inferior capsule equator, and the nucleus was dislocated downwards. Spontaneous lens capsule rupture in HMSC is extremely uncommon, especially nowadays, and has been seldom documented. In HMSC, the lens fibers imbibe fluid and get increasingly hydrated. The weight of the advanced nuclear cataract along with posteriorly directed pressure may lead to a spontaneous rupture and nucleus dislocation. The extremely hard nucleus in the presented case may suggest hypermature cataract as the cause. CME developed before and after surgery. Clinical CME is reported in up to 28% of eyes after PPV for dropped lens fragments, with a worse prognosis if the fragments were nuclear due a stronger inflammatory response or a more traumatic vitrectomy (5). Lens epithelial cells increase the synthesis of prostaglandins and cytokines, which can more easily diffuse to the macula in vitrectomized eyes. Also, CME development is reported in 46% of aphakic patients (6). The best timing of PPV for dropped nucleus remains unclear. Some studies suggest early PPV, while others report similar outcomes and complication rates regardless of the time of PPV (7). The disease course in the presented case suggests possible autoimmune reaction to lens proteins and the importance of early removal of the dropped nucleus. The rate of spontaneous closure of idiopathic FTMH hole varies from 2.7 to 6.2% (8). The closure usually happens in smaller holes (under 250 microns) within two months after the first presentation (8). Kumagaia et al (9) showed that the mean time from surgery to hole formation in 47 patients who developed FTMH after PPV was 20.4 months, with no spontaneous closure. After re-operation, the closure rate was 68%. Zhang et al (6) reported three cases of FTMH development in vitrectomized eyes within the first seven postoperative months, with spontaneous closure within two months after the onset of symptoms. The size of the holes was under 189 microns. Before FTMH formation, all patients had documented lamellar holes or epiretinal membranes. Similar reports (6,9,10) indicate that the presence of a mild epiretinal membrane (ERM) with a small FTMH may even be favorable for spontaneous closure. In our patient FTMH formed three months after successful vitrectomy for dropped nucleus. Hole formation was preceded by the formation of CME, ERM, and lamellar hole, and extremely high intraocular pressure, which could not be regulated by topical and oral therapy. The reason for such high IOP after the nucleus removal remains unclear. Both CME and high intraocular pressure might have been caused by proinflammatory factors, but our patient had no signs of inflammation either pre- or postoperatively. Spontaneous FTMH closure in our patient started two months after the first appearance (5 months after the vitrectomy) and lasted almost one year. Simultaneous to hole closure, IOP decreased to normal levels with topical anti-glaucomatous therapy. A clear lens capsule and dropped nucleus in a quiet eye with normal BCVA, which rapidly developed into intractable secondary glaucoma, CME, and FTHM, are an unusual finding. Uneventful surgery and non-steroidal anti-inflammatory drugs and steroid treatment were followed by spontaneous recovery, IOP normalization, and macular hole closure, but VA never completely recovered. Such disease course has not been previously described and suggests possible autoimmune reaction to lens proteins and the importance of early removal of the dropped nucleus.
